# “I Just Went for the Screening, But I Did Not Go for the Results”. Utilization of Cervical Cancer Screening and Vaccination among Females at Oyibi Community

**DOI:** 10.31557/APJCP.2021.22.6.1789

**Published:** 2021-06

**Authors:** Evans Appiah Osei, Amertil P Ninon, Judith E Gaogli, Ezekiel Oti Boadi

**Affiliations:** *School of Nursing and Midwifery, Department of Nursing, Valley View University, Ghana. *

**Keywords:** Utilization, cervical cancer, screening, vaccination, females

## Abstract

**Background:**

Cervical cancer screening and vaccination practices is reported to have low coverage in most developing countries. It has been reported that most women are aware of cervical cancer screening and vaccination worldwide. Nevertheless, the rate at which women participate in cervical cancer screening and vaccination was found to be low both locally and internationally. Consequently, in sub-Saharan Africa, cervical cancer screening programs have poor coverage. The aim of this study was to explore the practices of cervical cancer screening and vaccination among females at Oyibi community.

**Methods:**

The researchers employed a qualitative exploratory design to recruit 35 participants put into five Focus Group Discussions (FGDs). Five FGDs were formed with seven (7) members in each group. The members were purposely recruited. The sample size was based on data saturation. Data was retrieved using a semi-structured interview guide. The researchers served as moderators in the group.

**Results:**

Two (2) main themes with Eight (8) subthemes were generated from the data analysis. The themes were; (cervical cancer screening and vaccination practices), and (perceived benefits of cervical cancer screening and vaccination). The subthemes that emerged were as follows: types of cervical screening and vaccination done by participants, experiences during cervical cancer screening, experiences during cervical cancer vaccination, decision to go for cervical cancer screening and vaccination, willingness to recommend cervical cancer screening and vaccination to other women, early detection of cervical cancer through early screening, benefits of cervical cancer vaccination, and willingness to receive cervical cancer vaccine. The study also revealed that most of the women who had done the screening and vaccination were young (19-29 years).

**Conclusion:**

The results from the study indicated that the participants’ utilization of cervical cancer screening and vaccination were poor although they were conscious of the benefits of cervical cancer screening and vaccination and were willing to recommend it to their relatives and their loved ones.

## Introduction

Cervical cancer screening and vaccination practices are reported to be low in most developing countries. Most studies have ascertained that even though most women are aware of the types of screening for cervical cancer , few have participated (Markovic, et al., 2005; Jassim, et al., 2018; Liebermann et al., 2020 ). For instance, the study of Bakogianni et al (2012) among 472 female students ascertained that although majority of the participants (94.07%) knew about the Pap test, only 44.82% of the participants had been screened and vaccinated against cervical cancer. The major source of information about cervical cancer screening and vaccination identified by authors included the media, relatives, friends, and health workers (Ezem, 2007; Awodele et al., 2011). Majority of the participants in a study indicated that they received the vaccine after their first sexual intercourse (Bakogianni, et al., 2012). The reasons cited by some females for not screening and vaccinating included lack of appreciation of the importance of screening, feeling of embarrassment, fear and the attendant high cost (Makwe and Ihuma, 2012). In South Africa, a study revealed that, only 16 (9.8%) participants have had a Pap smear test done of which 11 (69%) knew their result (Hogue et al., 2014). A point worth noting is that, even though awareness and knowledge of cervical cancer were high among staff, their patronage of screening were low (Owoeye and Ibrahim, 2013)

Cervical cancer is a type of cancer which affects the cells of the cervix and is associated with the Human Papilloma virus (HPV) (Brisson, and Drolet, 2019). There are several types of HPV but the two common types linked to cervical cancer are type 16 and 18 (Arbyn et al., 2020 ; Vu et al., 2013). Cervical cancer screening is a form of analysis to detect HPV and precancerous cell to aid in reducing cervical cancer incidence and morbidities (Massad et al., 2013). Pap smear test is one of the types of cervical cancer screening where a sample is obtained from the cervix which is then smeared onto a labeled glass slide and fixed with 95% ethyl alcohol in a jar for analysis (Sachan et al., 2018). 

In sub-Saharan Africa, cervical cancer screening programs have poor coverage (Irabor et al., 2017). For example, it has been found that cervical cancer screening coverage in sub-Saharan Africa ranges from 2% to 20% in the urban areas and 0.4 to 14% in the rural areas (Louie et al., 2009). Sixty to eighty percent (60 - 80%) of women who develop cervical cancer in sub-Saharan Africa live in rural areas with no opportunity of taking part in a cervical screening (Irabor et al., 2017). Also, cervical cancer screening programs have been found to have a better coverage among those of higher socio-economic class which still puts the people of sub-Saharan Africa among those who have poor coverage of the screening programs and vaccination utilization (Irabor et al., 2018).

Regarding Human Papilloma Virus (HPV) vaccines, it is increasingly becoming available in the developing countries, but the cost is prohibitive for most people living in low and middle-income countries (Songane et al., 2019). The vaccine is recommended for girls prior to their sexual debut yet the HPV vaccine has not yet been deployed in the National Program on Immunization (NPI) in countries in Africa including Nigeria (Sadoh et al., 2018: Mutiu et al., 2019). Evidence suggests that in most developing countries, especially Ghana, there is inadequate infrastructure and quality control (Bobdey et al., 2016). Hence, the researchers reported that high quality cytology screening may not be feasible for wide-scale implementation thereby contributing to the high incidence of cervical cancer in developing countries. 

Despite the fact that the HPV vaccine has been authorized for use in Ghana and is available in some private and public health care centers, cervical cancer screening rates in urban and rural settings in Ghana are low thus 3.2% and 2.2% respectively (Williams and Amoateng, 2012). Cervical cancer screening among women in Ghana is very poor since those who undergo cervical cancer screening delay in looking for treatment until the point where their cervical growth tumors may have metastasized (Williams et al., 2013). It is shown that cervical cancer screening in many hospitals in Accra, especially the Ridge hospital are Papanicolau (Pap) smear and visual inspection of the cervix with acetic acid (VIA) (Sanghvi et al., 2008) which help in early detection of cervical cancer. 

In Ghana, some women have the intention and willingness to receive HPV vaccine due to their perceived benefits (Juntasopeepun et al., 2011). Despite the availability of cervical cancer screening tools in the country, including those that are appropriate for low resource setting, the rate of preventive cervical cancer screening remain extremely low among women in Low and Middle Income Countries (LMICS), hence, affects the use of vaccines (Williams et al., 2018). It can therefore be inferred that a positive attitude towards the cervical cancer vaccination and screening increase the chances to be screened and also receive the vaccine (Kang, and Moneyham, 2011).

*Purpose of the study*

The purpose of this study was to explore the practices of cervical cancer screening and vaccination among females at Oyibi Community.

## Materials and Methods

*Methodology*

In this study, a qualitative exploratory design was employed. Qualitative research is a form of inquiry that studies individuals in their natural settings and helps to interpret a phenomena in terms of the meanings people bring to them (Aspers and Corte, 2019). A focus group discussion was used in collecting data from participants. This was used because the researchers were interested in exploring the varied opinions of the women on cervical cancer screening and vaccination. 

A focus group discussion was used to gain an in-depth understanding of cervical cancer screening and vaccination from participants’ point of view(Nyumbaet al., 2018). The researchers served as facilitators during the data collection. The target population of this study included women living in the Oyibi community who were 18 years and above since women of such age range were at risk of cervical cancer because they are sexually active. The study included women between the ages of 18 to 65 years who could express themselves in Twi and English and were willing to participate in the study.

The research setting is Oyibi located in the Greater Accra Region of Ghana. It is one of the rural communities in the G.A of Ghana and no study has looked at cervical cancer screening and vaccination in this group. Finally, this community has no cervical cancer screening center close to them.Neither do they have a hospital. 

Purposive sampling was used to recruit all participants who met the inclusion criteria and were willing to engage in the FGDs to provide necessaryinformation to ensure credibility of the study. Sample size was based on data saturation. Five (5) FGDs were held with seven (7) members in each group. Hence, the sample size for this study was 35 participants. All the 35 participants completed the interviews with none opting out. Interviews were conducted from one group to the other till no new responses were retrieved (Data saturation). Ethical clearance was obtained from the Dodowa Health Research Center Institutional Review Board (DHRC- IRB 31/03/20) before the data collection. The clearance letter from the ethical board was submitted to the assembly man and elders of the Oyibi community who gave their permission for entry into the community. The researchers contacted various females from the selected community in various locations and gatherings such as churches, weddings, marketplaces and houses within the community. The researchers established rapport by introducing themselves to the participants before explaining the purpose of the study to them. The benefits participants stood to gain from the study was also explained to them. The researchers scheduled the days for the data collection and a venue to suit participants’ availability. Moreover, all methods were carried out in accordance torelevant guidelines and regulations including the informed consent, voluntary participation, anonymity and confidentiality. The interviews were recorded with an audio tape. The researchers met participants in a private place to conduct the interviews ensuring that no other person gets access to the recorded data. The participants were asked to use numbers to identify themselves in the group instead of their original names to conceal their identity. They were also informed about some “dos” and “don’ts” such as “Not interrupting others when they are sharing their views, allowing each member to share their views, not arguing with members of the group but can disagree where appropriate and not disclosing any personal information given during the discussion or making mockery of a group member after the discussion. The interviews were conducted by all the authors in English since all the participants speakand understand English. Focus group interviews lasted for 45-60 minutes. Data was collected over a period of 6weeks. The participants were congratulated by the researchers after the interviews. 

A semi-structured interview was used by the researchers who served as moderators to collect data from the members of each FGs. The interview guide consisted of open-ended questions with probes for further clarifications. The interview guide consisted of demographic characteristics of participants as well as questions based on the objectives of the study which were as follows: practices, willingness to screen and vaccinate and perceptions on cervical cancer screening benefits. The tool was carefully designed by the researchers and reviewed by all researchers. It was also given to other nursing researchers to peer review. The tool was pretested among two females in Malejor who have similar characteristics as the women in Oyibi. All interviews were done once. The recorded interviews were transcribed and saved on a personal laptop and it was secured with a password known only to the researchers. This was to ensure the safety of the interviews in case the laptop was stolen or became faulty.

*Statistical Analysis*

Data was analyzed using content analysis. Content analysis has been defined as a systematic way of compressing several words into fewer content categories based on explicit rules of coding (Stemler, 2000). It allows researchers sift through large volumes of data. The analysis was done after each FGDs. Data collection and data analysis were done concurrently. The audio taped data were played by the researchers and typed into a word document saving them with FGD numbers (1-5). The researchers played and listened to each discussion over and over again to familiarize themselves with the data which were transcribed verbatim. Each transcript was read and re-read to understand what participants said and to contact participants for clarification where appropriate. The researcher then coded the data by reading through and giving meanings throughout the transcripts by representing it with two (2) to four (4) words. Similar meanings were put together. Themes which were formulated for each group were written down and grouped based on patterns or relationships amongst them. In all, two (2) themes emerged and eight (8) subthemes were formulated based on theobjectives of the study.

The methodological rigor was maintained to ensure the validity and reliability of the findings. This ensured that+ findings and the processes for conducting the data were s trustworthy. The trustworthiness was maintained by ensuring the following weremaintained: credibility, transferability, dependability, and confirmability (Bittlinger, 2017).

## Results

*Socio-demographic characteristics of the participants*

Thirty-five (35) participants constituting five (5) Focus Group Discussions (FGD) were interacted with to obtain necessary data for the study. Each FGD consisted of seven (7) participants. The group consisted solely of o women from Oyibi Community within the Kpone-Katamanso District in the Greater Accra Region of Ghana.

The findings of the study revealed that majority of the participants (69%) were single whilst few of them, constituting 11 (31%) were married for 5years and above. Concerning the age of participants, the results revealed that majority of the participants were within the age of 19 -29 years with few above 40 years. Thus, the least age recorded was 19 and 60 years as the highest age recorded. The study again revealed that majority, that is, 15 participants of the respondents had Secondary education (57.1%) and Tertiary education 13 (37.1%) with 7 making the least participants having basic education background (20%). Majority of the participants 33 (94.3%) were Christians while a few of them (5.7%), were Muslims with diverse cultural backgrounds from the Volta region, Ashanti region, Eastern region, Greater Accra region, Northern region, and Western region. The rest of the demographic data is presented below.

Two themes emerged from this study which were cervical cancer screening and vaccination utilization by women and cervical cancer vaccination effectiveness and cost. 

*Utilization of Cervical Cancer Screening and Vaccination*

This theme presents practices of cervical cancer screening and vaccination among women. The five (5) sub-themes which emerged were the types of cervical screening and vaccination done by participants, experience during cervical cancer screening, experiences during cervical cancer vaccination, decision to go for cervical cancer screening and vaccination, and willingness to recommend cervical cancer screening and vaccination to other women.

*The types of cervical screening and vaccination done by participants*

Analysis of the data collected revealed that few of the participants had undergone cervical cancer screening 2 (5.7%) (20 and 25 years) and vaccination 1 (2.9%) (28 years). The following quotes provide details of the above results:

“*I have done the pap smear at work, but I haven’t taken the vaccine. It was free at my workplace, so I just went to screen to know if I have it since I am young. However, they didn’t tell me to come for the vaccine because I didn’t go for the report. They did not also do any follow up to ask why I didn’t come for the results although they took my number*”(p.20).

“*Yes, I was told the screening is called pap smear. I went to screen last year when it was the cervical cancer screening month. It was free as at that time. After I was told it was a negative, I paid for the vaccine and they administered it to metwice. I did that because I married at the age 23 and two years now, I have not been able to conceive so I wanted to be sure nothing was wrong with my cervix*’’ (p.25).

Few participants who refused to go for the cervical cancer screening and vaccination stated that theywere virgins hence not legible for screening:

“*No, I have not gone to screen or take the vaccine. Per the information I had, I was told that only those who are sexually active could be screened of which I am not. I am a virgin so there is no need going to do it. I might do it in the future*” (p.15).

Other participants did not go for the cervical cancer screening and vaccination because they lacked knowledge on its importance:

‘’ *I haven’tbeen screened and vaccinated because I don’t think it is necessary to screen because I don’t have the disease. Even at the age of 45years, I am still strong. I am saying this because I am not sick, and I think it is not necessary. I don’t also know the types of cervical cancer screening and vaccines given, so why should I screen and vaccinate*?’ (p.16).

“*I cannot be affected by this cancer because I am 50 years plus and I have not been hospitalized before and in my family no one has had this condition*’ (p.35).

*Experiences during cervical cancer screening*

The study discovered that only few of the participants had done the cervical cancer screening. Various interesting and insightful experiences reported by participants during cervical cancer screening were as follows:

“*Oh, I did the pap smear at my work place, so I was a bit comfortable with the environment. The procedure itself was uncomfortable though painless. When I got to the room, I was asked to lie down on the bed and put my legs on a leg support. I was alone with the nurse. The nurse inserted an equipment into my vagina to take fluid for investigation*’’(p.6).

‘’ *We entered a room.Then the nur explained how it was going to be. I was okay because there was no man there. She assisted me to lie on the bed and raised my legs and put them on a leg support.After that she put some cloth on my thighs then I felt her putting something in my vagina, but it wasn’t painful. It was normal, however I felt something brushing inside like twice*’’ (p.20).

*Experiences during cervical cancer vaccination*

A significant experience revealed in this study was pain associated with the cervical cancer vaccination since it is injected. Hence, the study found that only one (2.8%) participant had received the vaccine for cervical cancer even though few of them had done the screening

‘’*After I paid for the vaccine, I was asked to wait in a room. The nurse then came in with some needles and the vaccine in a small bottle. She told me she wouldinject my upper arm and it would feel uncomfortable a bit. She took the vaccine with the needle and then cleaned the area with cotton and injected it, even though it felt painful she was soon done*’’ (p.25).

Other participants shared their experiences on how the vaccination might be even though they had not been vaccinated:

‘’ *I don’t know if it’s painful or not even though I know it’s an injection. I didn’t go for the vaccine even though I have done the screening once because I do not like injections*” (p.20).

‘’ *I have heard of the vaccine but I don’t know if it is an injection or poured into the mouth like the vitamin A vaccine, so I cannot tell how it feels like*’’ (p.1).

‘‘*No please, I have no idea about the vaccine and how it’s like. I didn’teven know that cancer has a vaccine that prevents it. If my grand-daughter was to be around she would have been able to tell you’*’ (p.4).

*Decision to go for cervical cancer screening and vaccination*

The results suggest that more than half of the participants, 20 (57.1%) were willing and eager to receive cervical cancer screening and vaccination ifthey have the opportunity;

“*I will be very glad to go for the cervical cancer screening and vaccination since I am aware of the cervical cancer screening and vaccination now but I will be happy to get it done soon*’’(p.2).

“*Yeah, definitely the idea came to mind one day. I might go for the screening and get a review of my reproductive organs to know if everything is okay soon*‘’.(p.6)

“*I would like to screen and get vaccinated against cervical cancer soon. I had the intention of getting myself screenedabout a year ago, but I have been too busy with work to take time off to do so*’’(p.17).

However, few participants didn’t see the need to screen and get vaccinated because they were advanced in age;

‘’* No, I didn’t get screened and vaccinated when I was young because I didn’t know about the condition, and also I don’t have any intention to get vaccinated now because I am old and my husband is is no more alive so I will rather recommend it to the young ones who are sexually active*’’ (p.8).

“*I have not screened and vaccinated. In fact, I have no reason to be screened because I am not sick, I am healthy. It’s when you are ill that you visit the hospital to get treated.S I am well, so why should I go?*” (p.29).

*Willingness to recommend cervical cancer screening and vaccination to other women*

Further discussion with the participants on the above subject matter revealed that they are ready to recommend to all women including their friends, families and church members to screen and vaccinate against cervical cancer:

“*It’s a good initiative, so I will recommend it to other people to go and bescreened. I will tell my friends, church members and family about it since I now know about it. Knowing about this disease and getting screened for it comes with huge benefits, so I will take it upon myself as a duty to tell all women in my church it*”. (p.1)

“*I will recommend it to all women to get screened for it and also to get the vaccine because sex is common among the young women of today, so it will be good if they know their status*” ( p.24).

Participants revealed that they are not aware of any relative or friend who had done the screening and hence, recounted that they were going to recommend the screening and vaccination to their friends and relatives:

“*I don’t know of any relative and friends who have gone to be screened and also received the vaccine, so I will tell my family members and friends about it when I go home*” (p.34).

“*No please, I don’t know of any family members or relatives that have done it. You know, when it comes to illnesses like this,it’s not something people would want to know, so I think that is the reason why they have not done it.however, I will tell them the importance of doing it, so that we all can go for the screening*”(p.31).

*Perceived Benefits of Cervical Cancer Screening and Vaccination*

The participants viewed the screening as beneficial. Three (3) subthemes emerged from this theme: early detection of cervical cancer through early screening, willingness to receive cervical cancer vaccine, cost effectiveness.

*Early detection of cervical cancer through early screening*

Interrogations with the participants on the above subject matter revealed that early detection of the disease helps in early treatment and prevention which eventually reduces the death rate of cervical cancer. In regard to the above findings, participants explained in the following words:

 “*I think if you go early to screen for cervical cancer and it is detected , it is good because early detection of the disease is key to prevent it. You waiting until the disease spreads to other parts of the body will be worse, so early detection is the best*” (p.28).

Few participants narrated that knowing it early will help one commence treatment early to prevent complications:

“*Yes, I say it helps to detect it early because if you go and get screened and it is positive you get treated and when its negative you get vaccinated to protect you, so both are good*”.(p.20)

‘‘*I believe that you going to get screenedand vaccinated will reduce the number of women who are infected with cervical cancer since the screening helps to detect cervical cancer. You can receive treatment when it is detected early. It will not only reduce the number of people having the disease but it will also reduce the death rate. It will help salvage the rest of the cervix that is not infected and treat the in time’*’ (p.35).

*Protection from cervical cancer*

Cervical cancer vaccination is one of the most important things to do after screening. The participants also recounted that vaccination for cervical cancer is a step in the right direction to preventing the disease. 

“*The vaccine gives lifetime protection when taken; you don’t have to worry about getting cervical cancer after screening and taking the vaccination*”. (p.4).

“*I believe that early screening and vaccination against cervical cancer will help to prevent this disease because after screening, if the result is negative, you will receive the vaccine that prevents you from cervical cancer. And as our people say prevention is better than cure*’’. (p.3).

However some participants believed that cervical cancer could not be prevented by receiving the HPV vaccine since cancers cannot be prevented:

‘*‘Cancers in general cannot be prevented with vaccines. I don’t know of any vaccine that prevents cervical cancer. When you get cervical cancer that is it. The only thing left is to go for treatment like chemotherapy and other medications*’’(p.33).

“*Vaccines for preventing cancer? I didn’t know there was a vaccine available for preventing cervical cancer because people are still dying of cancers. You just have to be mindful of your lifestyle, that is the best way to prevent it*’’ (p.24).

*Willingness to receive cervical cancer vaccination*

Participants revealed that after screening for cervical cancer, it is important to go for vaccination to protect oneself. The following quotes provides details of the results above:

“*I’ m willing to go for the screening and vaccination to protect myself from this deadly disease, even if I have contracted the cancer after being screened, I will be ready to be treated for the , and if I am negative too, I will go for the vaccination to help protect me from the cancer.* (p.13).

‘‘*In my case, I have gone to have cervical cancer screening already but I was reluctant to go for the vaccination but I think I am ready to go for the vaccination now*’’ (p.15)

‘‘*I have taken all my children for immunizations and they are all fine, so I am sure that this vaccine too will not cause any problem, so I will go for it whenever I am off duty*’’ (p.4)

**Table1:** Demographic Characteristics of the Participants

Details	Frequency (N=35)	Percent (%)
Age category of participants:		
19- 29 years	25	71.4
30-39 years	5	14.3
40 years and above	5	14.3
Marital status		
Single	24	69
Married	11	31
Years of Marriage		
0-4 years	24	68.6
5-10 years	6	17.1
Above 10 years	5	14.3
Educational backgrounds:		
Basic education	7	25.7
Secondary education	15	57.1
Tertiary education	13	17.2
Occupations of participants		
Student	10	28.6
Government/ Health Worker	9	25.7
Sales Persons/ Attendants	4	11.4
Self- employed/ Entrepreneurs	2	5.7
Hair Dresser	4	11.4
Seamstress	3	8.6
Unemployed	3	8.6
Religion of participants		
Christianity	33	94.3
Islamic	2	5.7
Cultural backgrounds		
Volta region	11	42.9
Greater Accra region	6	17.1
Ashanti region	4	11.4
Eastern region	3	8.6
Northern region	6	17.1
Western region	5	14.3
Parity of participants	15	
No child	8	42.9
1 child	8	22.9
2 children	4	22.9
3-5 children		11.4
Total	35	100

Source, Filed Survey Data (2020).

## Discussions

*Utilization of Cervical Cancer Screening and Vaccination*

Results of the present study revealed that just a few participants had undergone cervical cancer screening and vaccination thus 2 (5.7%) and 1 (2.9%) respectively. Participants who had undergone screening recalled pap-smear as the test done. However, participants who had received the vaccination could not recall the name of the vaccine that was administered. The present study further reveals that some of the participants did not go to be screened and vaccinated on the basis that they were virgins, hence, not legible for screening. This finding is in consonance with a study by Aniebue and Aniebue (2010) among 394 female university students in Nigeriawhich unveiled that, about 23.1% identified the Pap smear as a screening test type and only 5.2% of respondents had ever been screened. The findings also supported a study done in Ghana which discovered that The majority of women(97.7%) had never heard of the Pap smear test before (Ebu et al., 2015). Nevertheless, the study was in contrast to Harper, and DeMars’ (2016) study in Canada which showed that, Cervarix and Gardasil9 were some vaccines mentioned by participants.

The few participants who had undergone cervical cancer screening shared their experiences during screening. These participants recounted that, the screening was not painful during the procedure, but they were rather uncomfortable because of the way they were placed on the table to be examined and the equipment that was inserted into their vagina. Findings also recorded that, some participants were comfortable during the screening due to familiar environment and the absence of male nurses or doctors. The present study is in likeness to a study conducted by Rositch et al., (2012) who discovered that some women reported a low level of physical discomfort during Pap smear collection. In addition, over 80% of women reported that they would feel comfortable using a self-sampling device (82%) and would prefer at-home sample collection (84%). This present study’s finding was in contrast with a study where 26.47% of respondents were married but none of them had undergone screening test with the belief that it would be painful (Pegu et al., 2016). A similar study in Ghana also revealed that only 15 of the participants (8.5%) had undergone Pap smear for cervical cancer screening due to poor knowledge about it (Adanu, 2002). 

*Experiences during cervical cancer vaccination*

A significant experience revealed in this study was pain associated withcervical cancer vaccination. The study found that, only one (2.8%) participant had received the vaccine for cervical cancer even though a few of them had done the screening. The participant who had received the cervical cancer vaccination described the process as a bit painful although not as painful as she thought it would be. She felt the needle pricking her skin for just a brief period. This implies that the experiences of females on cervical cancer vaccination is mainly subjective since others may perceive it differently. Similarly, a study conducted in North Carolina reported that pain from HPV vaccination was commonly reported by parents but was less frequent compared to other adolescent vaccines and did not appear to affect vaccine regimen completion. These findings may be important to increase HPV vaccination coverage since women perceive it as less painful. (Hudson et al., 2016). Moreover, a study done in Ghana ascertained that the main concern of the vaccination was ensuring safety during the administration rather than the pain as identified in this study (Coleman et al., 2011).

Regarding the decision to go for cervical cancer screening and vaccination, the study discovered that, more than half of the participants 20 (51%) were willing and eager to receive cervical cancer screening and vaccination should they have the opportunity. In relation to the present study, a study conducted in Nigeria revealed that majority of the participants (62.5%) demonstrated readiness to be screened and vaccinated against cervical cancer (Eze et al., 2012). 

About participants’ willingness to recommend cervical cancer screening and vaccination to other women, the study established that majority of the participants in the study were willing to recommend to all women including their friends, families and church members to be screened and vaccinated against cervical cancer since they believed most youth were into early sexual debut which was identified to increase participants’ risk in acquiring cervical cancer. The participants also revealed that, they were not aware of any relative or friend who had done the screening.They stated they were going to recommend the screening and vaccination to their friends and relatives. This finding was surprising because majority of the study participants had not been screened and vaccinated but were willing to recommend it to others. The findings of this current investigation are also consistent with those of Adejuyigbe et al., (2015), who did a study among medical students of the University of Lagos and found that most of the respondents supported vaccination of adolescent girls (65.7%) and were willing to recommend vaccination to colleagues/friends (82.1%) and to future clients (80.0%). 

*Perceived Benefits of Cervical Cancer Screening and Vaccination*

Early detection of cervical cancer was one of the benefits enumerated by the study participants. Few of the participants who knew about cervical cancer screening and vaccination were of the notion that, screening for cervical cancer aids in the early detection of cancer’s hidden warning signs long before symptoms appear and thus helps one to commence treatment early. The findings of this present study are consistent with that of Wang et al., (2019) who discovered that early cervical cancer screening and vaccination reduces cervical cancer cumulative incidence, increases life span, reduces cost of treating cervical cancer and improves the quality of life . Similar to the present study findings, Ibekweet al.(2010) in South Africa concluded that, majority (87%) were of the belief that cervical cancer screening is important, while 75% indicated that screening could find changes in the cervix before full cancer arises and that when cervical cancer is detected earlier, it can be easily treated.

Majority of the participants in this present study acknowledged the money spent in preventing cervical cancer was less than the money involved when treating cervical cancer, especially when the condition advances.Thus, it is perceived to be cost effective. This implies that the cost of preventive intervention of cervical cancer ischeaper, effective and beneficial than cost involved at the onset of the cervical cancer. However, these findings were surprising since majority of the participants had not been screened and vaccinated against cervical cancer. A study was in accordance with these findings where it was discovered that majority of the respondents (76%) used in a study in Ghana were willing to pay at least something for screening and vaccination because they were aware of the cost effectiveness of these services (Opoku et al., 2016).

In conclusion, the results from the study indicated that the participants’ utilization of cervical cancer screening and vaccination were poor, nevertheless, they were conscious of cervical cancer screening and vaccination and were willing to recommend it to friends, relatives and loves ones.

*Implications*

In nursing practice, the study revealed that cervical cancer screening and patronization rate among women who partook in this study was low, hence healthcare professionals should find ways of sensitizing women during healthcare delivery to encourage more women to partake in the screening. Moreover, nurses should prepare women who are being booked for the screening and vaccination to help allay the fears and anxieties that they go through. Healthcare professionals should follow up on the women who come for screening to motivate them to go for vaccination. Durbars and seminars should be organized in various rural communities in Ghana by health care professionals, hospitals and N.G.Os in the country to sensitize women on cervical cancer screening and vaccination to help clear misconceptions.

*Recommendations*

The government should formulate policies to help reduce cost for screening and vaccination. The NHIS should absorb the cost to help improve screening and vaccination coverage. 

*Ministry of Health (MoH)*

• The Ministry of Health should lobby government for funds for the inclusion of the cost of cervical cancer screening and vaccination in the NHIS. 

*GHANA HEALTH SERVICE (GHS)*

• Ministry of Health should establish more screening centers a screening center close to the rural community where the study was conducted to motivate more women in this area to patronize screening for cervical cancer and for vaccination.

• They should also make screening and vaccination services available and accessible nation-wide.

*PRACTICING NURSES*

• They should make out time to explain the screening procedure to the client and the possible discomfort that might occur.

• They should also take the telephone numbers of the clients that und ergo screening in order to call them when the results are ready.

•Health care professionals should conduct more research in this field to help find innovative ways to improve screening and vaccination practices.

Limitations

• The study might have revealed more robust findings if a mixed method was done. Hence it is recommended that other researchers consider doing a mixed method in this area.

## Data Availability

All data generated or analysed during this study are in the custody of the researchers and will be made available upon request.
